# Kv4.3 Modulates the Distribution of hERG

**DOI:** 10.1038/s41598-017-17837-6

**Published:** 2017-12-19

**Authors:** Xiao-Jing Zhao, Chao Zhu, Liu-Yang Tian, Yi-Cheng Fu, Yu Zhang, Xi Chen, Yun Huang, Yang Li

**Affiliations:** 1 Department of Cardiology, General Hospital of Chinese People’s Liberation Army, Beijing, China; 2 Department of Gerontology, Union Hospital, Tongji Medical College, Huazhong University of Science and Technology, Wuhan, China

## Abstract

This study examines the interaction between hERG and Kv4.3. The functional interaction between hERG and Kv4.3, expressed in a heterologous cell line, was studied using patch clamp techniques, western blot, immunofluorescence, and co-immunoprecipitation. Co-expression of Kv4.3 with hERG increased hERG current density (tail current after a step to +10 mV: 26 ± 3 *versus* 56 ± 7 pA/pF, *p* < 0.01). Kv4.3 co-expression also increased the protein expression and promoted the membrane localization of hERG. Western blot showed Kv4.3 increased hERG expression by Hsp70. hERG and Kv4.3 co-localized and co-immunoprecipitated in cultured 293 T cells, indicating physical interactions between hERG and Kv4.3 proteins *in vitro*. In addition, Hsp70 interacted with hERG and Kv4.3 respectively, and formed complexes with hERG and Kv4.3. The α subunit of I_to_ Kv4.3 can interact with and modify the localization of the α subunit of I_Kr_ hERG, thus providing potentially novel insights into the molecular mechanism of the malignant ventricular arrhythmia in heart failure.

## Introduction

Cardiac repolarization is driven by potassium (K^+^) currents flowing through K^+^ channels. More specifically, the transient outward potassium current (I_to_) is responsible for the early repolarization (phase 1) of the cardiac action potential^1,2^, while the rapid (I_Kr_) and slow (I_Ks_) delayed rectifier potassium currents are responsible for the rapid repolarization phase (phase 3)^3,4^. First proposed by Roden in 1998^5^, “repolarization reserve” describes that the inhibition of a current, for example I_Kr_, will not lead to failure of repolarization. Instead, another current, I_Ks_, which remains activated, will act and prevent excessive repolarization delays^6–8^. This compensatory potential is lost when I_Ks_ is inhibited simultaneously, thus enhancing the effects of I_Kr_ inhibition and increasing the consequent risk of excessive repolarization delay and arrhythmias^6,7,9^. The “repolarization reserve” was then revised^10^ to that the loss of a single K^+^ current will not completely impair repolarization (and cause, for example, marked QT prolongation) because of redundancy of K^+^ currents; when one K^+^ current is dysfunctional, the other K^+^ current will increase to compensate so that the changes in action potential duration (APD) are minimized. This compensation requires delicate interaction between the different K^+^ currents.

A classic voltage-gated K^+^ channel (Kv channel) consists of four pore-forming (α) subunits that contain the voltage sensor and ion selectivity filter and accessory regulating (β) subunits^11,12^. For example, the co-assembly of α (KvLQT1) with β (minK) subunits forms I_Ks_ channels, while the co-assembly of α subunits encoded by the human ether a-go-go-related gene (hERG)^13^ and β subunits encoded by the KCNE2 gene product minK-related peptide 1 (MiRP1)^14,15^ are responsible for the formation of I_Kr_, although the contribution of MiRP1 to native I_Kr_ remains uncertain. There is a consensus that the pore-forming α subunits of the Kv4 subfamily, Kv4.3 (*KCND3*), encodes I_to_ channels^16^. However, β subunits have also been proposed to be part of myocardial I_to_ channels^17–19^. Delpon *et al*. suggested that the co-association of β subunits of MiRP1 with α subunits of the Kv4.3 contribute to the function of I_to_ channels^19^.

Studies show that different K^+^ currents interacted with each other through α-α subunits or α-β subunits. It is well established that the interaction between I_Kr_ and I_Ks_ is patho-physiologically relevant^20^. The prolongation of APD because of a reduction in I_Kr_ favors the activation of I_Ks_, which prevents an excessive repolarization delay, and thus suggests potential interactions between the α subunits of I_Kr_ and I_Ks_. In 2004, Ehrlich *et al*. demonstrated that the α subunit of I_Ks_ KvLQT1 could interact with the α subunit of I_Kr_ hERG to enhance hERG current densities and increase hERG-protein membrane localization^20^. Wu *et al*. also suggested that the α subunits of I_to_ Kv4.3 interact with the β subunits of I_Kr_ MiRP1, forming α-β subunits interaction, which is critical for the normal function of the native I_to_ channel complex in a human heart^21^.

During heart failure, electrophysiological and structural remodeling changes occur^22,23^. hERG and Kv4.3 mRNAs, and protein expression levels decrease^24^. I_Kr_ current and I_to_ current densities are reduced^25,26^. However, it has not been reported if the α subunit of I_Kr_ hERG interacts with the α subunit of I_to_ Kv4.3 during heart failure. Therefore, in light of the “repolarization reserve” theory, our present study aim to investigate the interactions between the α subunits of I_Kr_ and I_to_ using heterologous cell line co-transfected with hERG and Kv4.3.

## Results

### Effects of Kv4.3 on hERG current

**Figure 1:**
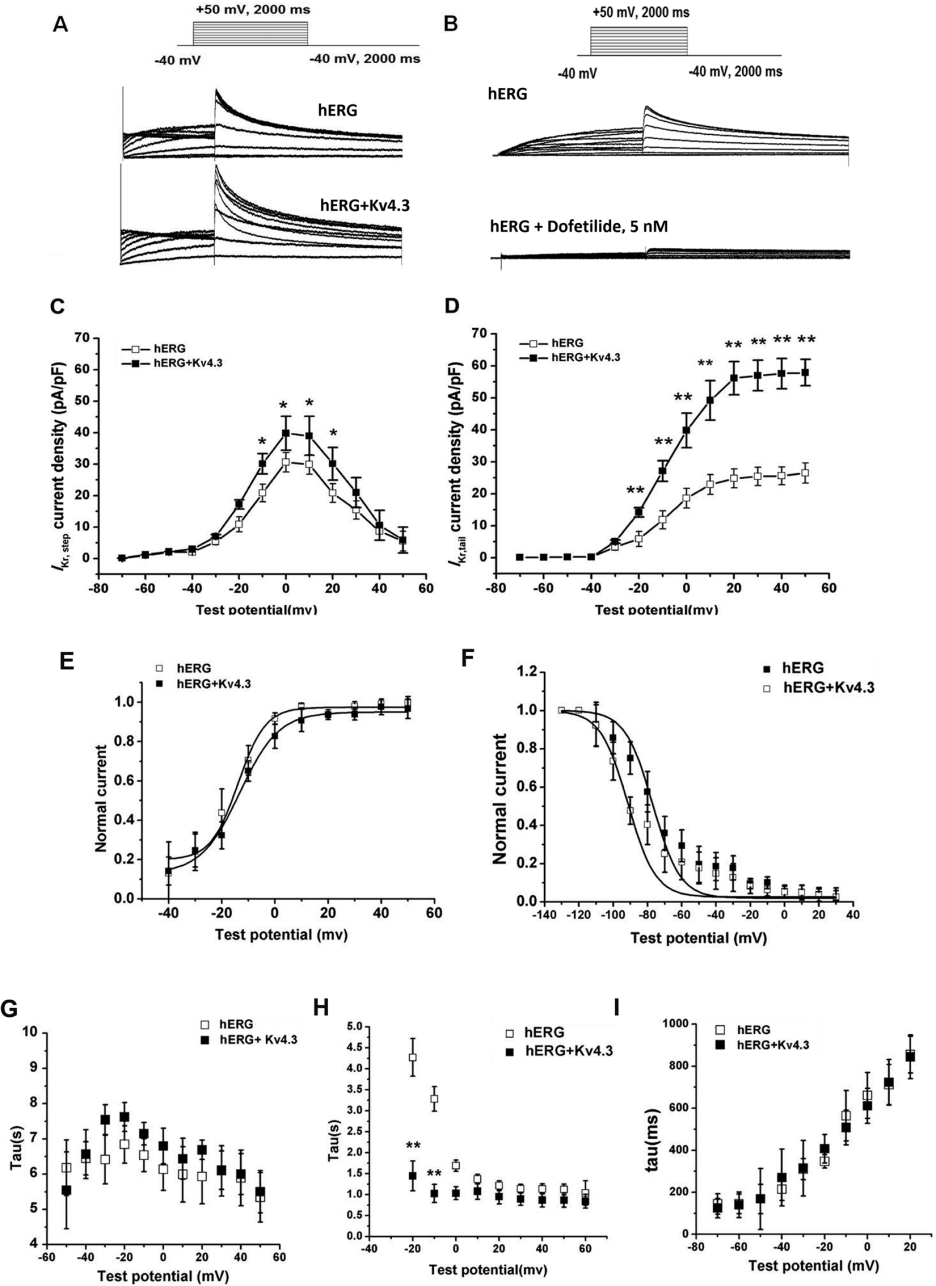
Co-transfection of Kv4.3 and hERG increases the hERG currents. A and B, recordings from 293 T cells transfected with hERG without (**A**) or with (**B**) Kv4.3 co-transfection (voltage protocol in the inset). Co-transfection of Kv4.3 and hERG resulted in significantly greater hERG tail currents (**C**) and step currents (**D**) than those transfected with hERG alone. The steady-state activation (**E**) and inactivation (**F**) in the cells transfected with hERG alone did not differ from cells co-transfected with Kv4.3. The activation time constants (**G**), the deactivation time constants (**H**) and the time constant of recovery (**I**) were unaltered by co-expression of Kv4.3. *p < 0.05; **p < 0.01; n = 15 and 20 for hERG and hERG + Kv4.3, respectively.

Figure 1A shows currents recorded from cells expressing hERG without and with Kv4.3 co-transfection. Incubating the cells with 5 nM dofetilide significantly reduced the recorded current, confirming that the currents recorded are hERG currents (I_hERG_, Fig. 1B). Co-transfection of Kv4.3 increased I_hERG_ densities, compared with cells transfected with hERG alone. Step (Fig. 1C) and tail (Fig. 1D) I_hERG_ densities were significantly larger in co-transfected cells (tail current after a step to +10 mV: 26 ± 3 *versus* 56 ± 7 pA/pF, *p* < 0.01)

There were no differences observed in the voltage dependence of activation of cells expressing hERG alone or co-expressed with Kv4.3 (V_1/2_ −13.89 ± 3.77675 *versus* −13.26 ± 1.27843 mV, n = 15 and 20, respectively, *p* = NS), (Fig. 1E), and in the steady-state inactivation voltage-dependence (V_1/2_ −93.57 ± 4.3758 for hERG *versus* −93.27 ± 4.0758 mV for hERG ±Kv4.3, n = 20, *p* = NS), (Fig. 1F). The activation time constants were unaltered by co-expression of Kv4.3 (Fig. 1G). However, the deactivation time constants upon repolarization to −50 mV after a 1.5 s step to +20 mV were smaller in cells co-transfected with hERG +Kv4.3 (4.25 ± 0.451 s, n = 18) versus those with hERG alone (3.33 ± 0.250 s, n = 12), (Fig. 1H). The time constant of recovery of cells expressing

hERG alone showed no significant difference when compared with cells co-expressed with hERG and Kv4.3 (Fig. 1I).

### Effects of hERG on Kv4.3 current

**Figure 2:**
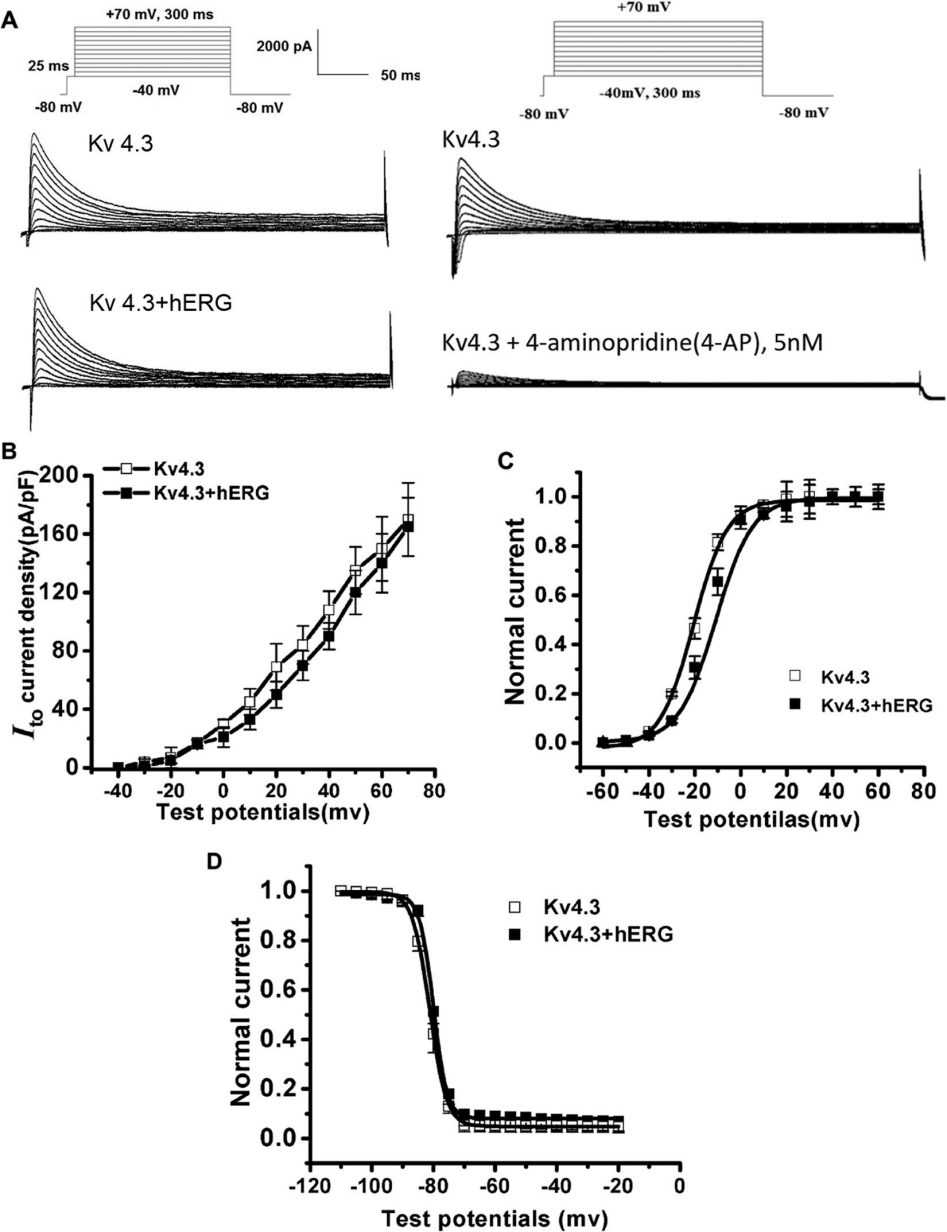
Co-transfection of Kv4.3 and hERG has no effect on the Kv4.3 currents. (**A**) Recordings from 293 T cells transfected with Kv4.3 with or without hERG co-transfection (voltage protocol in the inset). (**B**) The currents were the same in cells transfected with Kv4.3 or in cells co-transfected with hERG. No differences were observed in the steady-state activation (**C**) and inactivation (**D**) of cells transfected with Kv4.3 and Kv4.3 + hERG.

Since the expression of Kv4.3 affected I_hERG_, we next determine whether the reversed could also be true; *i.e*., whether expression of hERG alters I_to_ currents. Figure 2A shows representative recordings of currents from the cells transfected with Kv4.3 alone or both Kv4.3 and hERG. We also confirmed the currents recorded is I_to_ because the currents were significantly reduced by 5 nM 4-AP (Fig. 2A, the right panel). Co-transfection of hERG did not affect Kv4.3 current densities; there was no significant difference between those two groups (Fig. 2B). No differences in steady-state activation voltage-dependence were observed (*V*_1/2–20.86_ ± 3.10 for Kv4.3 *versus* −19.82 ± 4.18 mV for Kv4.3 + hERG, *p* = NS), (Fig. 2C). Similarly, steady-state inactivation voltage-dependence was unaffected (*V*_1/2_–80.86 ± 4.98 for Kv4.3 *versus* −79.82 ± 5.08 mV for Kv4.3 + hERG, *p* = NS), (Fig. 2D).

### Effects of Kv4.3 on hERG protein expression and localization

**Figure 3:**
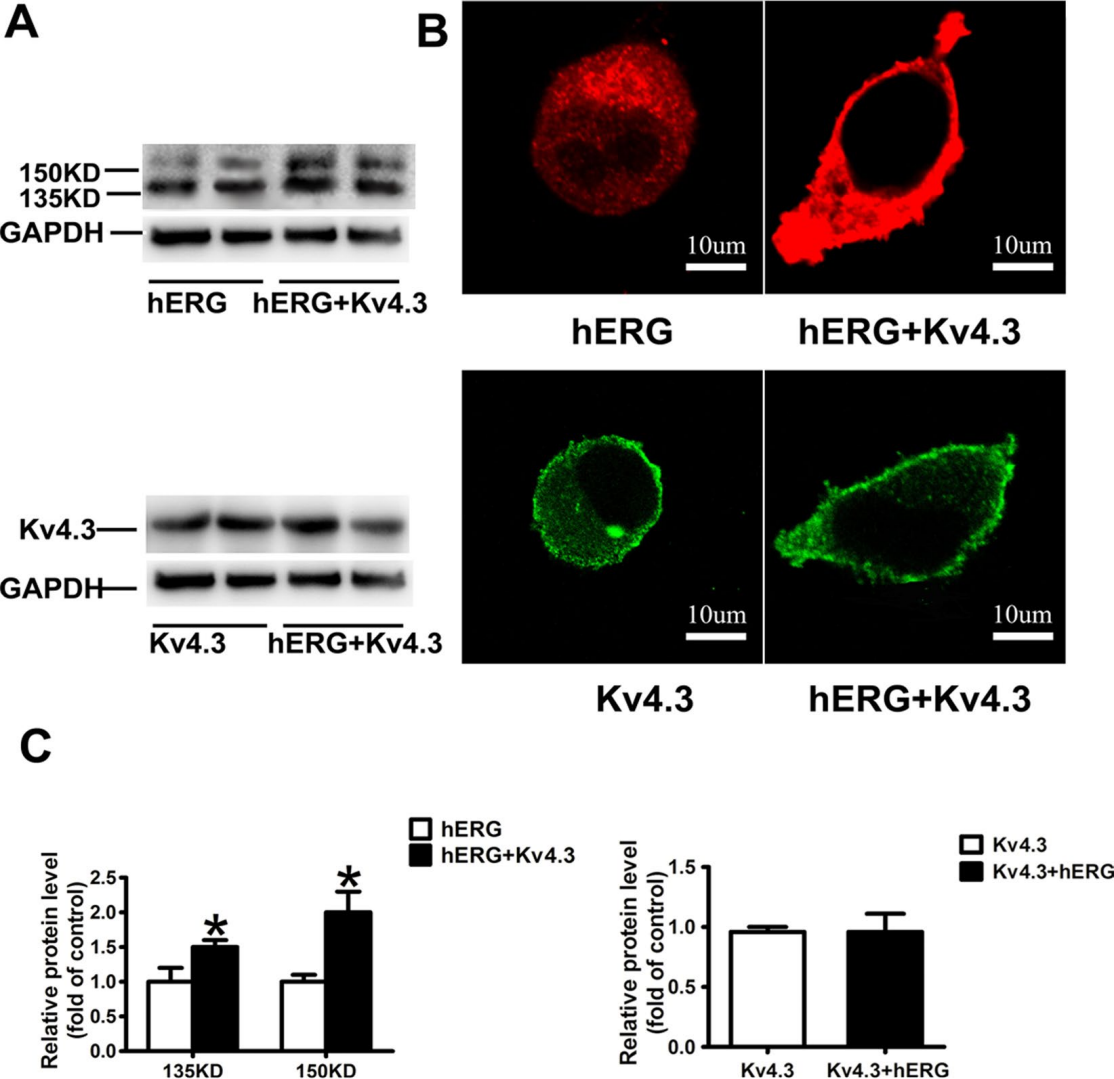
Co-transfection of Kv4.3 and hERG enhances the protein expression of hERG. The upper panel (A) shows the co-transfection of Kv4.3 increased the protein level of the fully glycosylated mature hERG (155 kDa). However, the lower panel (A) shows that the co-transfection of hERG did not increase the protein expression of Kv4.3. (**B**) Confocal microscope image of a cell transiently transfected with hERG or Kv4.3 alone (left) or co-transfected with hERG and Kv4.3 (right). The upper panel (B) Red represents immunofluorescence of secondary antibody to anti-hERG. Greater fluorescence was observed (right) at the whole cell compared with the left. The lower panel (B) Green represents Kv4.3 fluorescence signal (FITC-coupled secondary antibody). (**C**) Quantitative analysis of the data in (**A**).

The western blot showed that co-transfection of Kv4.3 increased the protein levels of the fully glycosylated mature hERG (155 kDa), but not the core-glycosylated, immature form (135 kDa). However, the protein expression of Kv4.3 was not altered by the co-transfection of hERG (Fig. 3A and C). Confocal imaging of cells transfected with only hERG displayed a strong hERG fluorescence in the cytoplasm; while cells co-transfected with hERG and Kv4.3 showed a homogeneous pattern of increased fluorescence throughout the cytoplasm and cell membrane. However, the distribution or density of Kv4.3 was not altered in cells co-transfected with Kv4.3 and hERG when compared with cell transfected with Kv4.3 only (Fig. 3B).

### Involvement of Hsp70 in Kv4.3-induced hERG expressions

**Figure 4:**
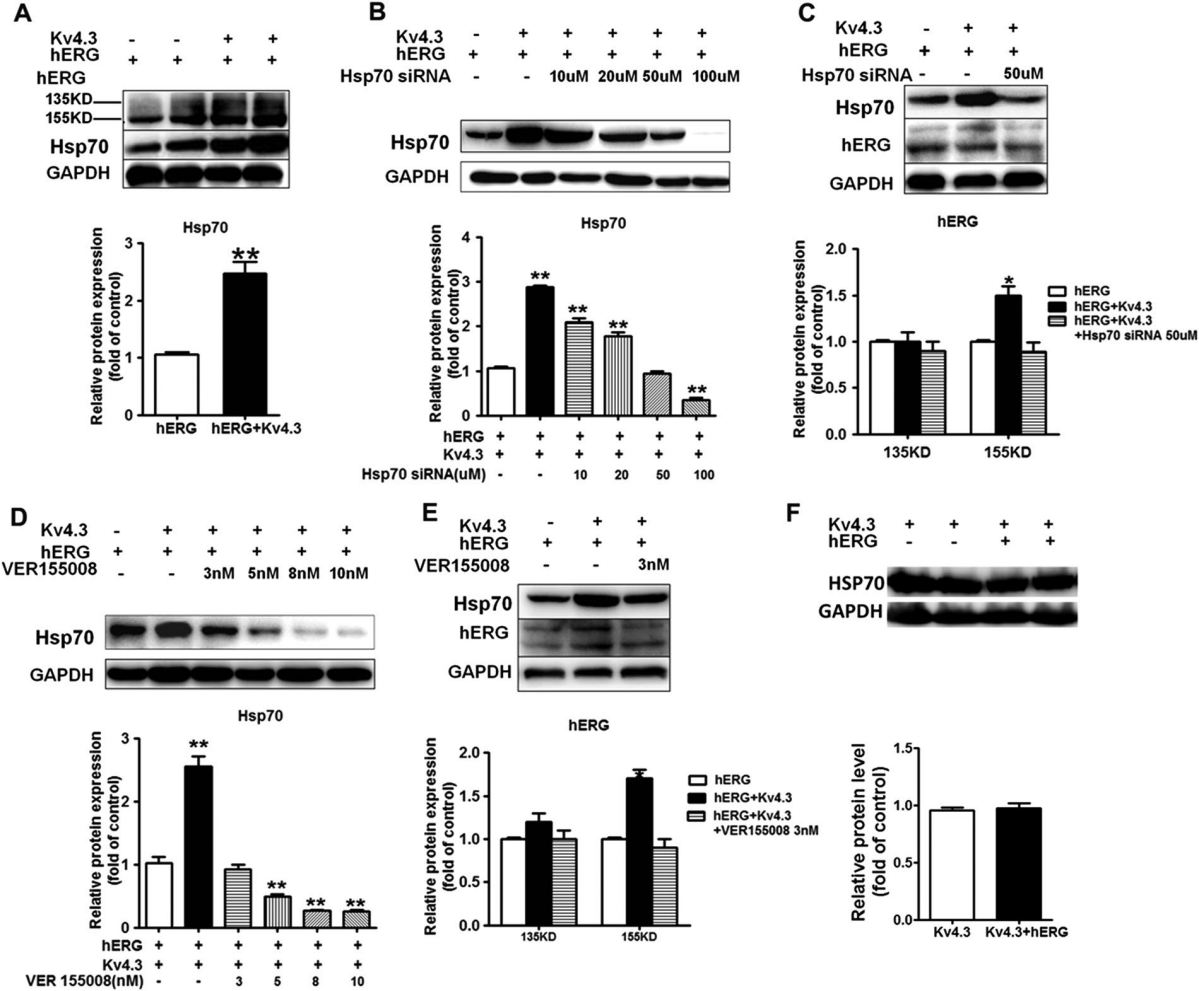
Hsp70 mediates the Kv4.3-enhanced hERG protein expression. (**A**) Co-transfection of Kv4.3 and hERG increased Hsp70 protein expression. (**B**) Cells co-transfected with hERG and Kv4.3 were preincubated with a dose of (10, 20, 50 and 100 μM) Hsp70 siRNA for 48 h. (**C**) The hERG protein expression levels in cells co-transfected with Kv4.3 and hERG preincubated with 50 μM Hsp70 siRNA were reduced to the same level as in cells transfected with hERG alone. (**D**) Cells co-transfected with hERG and Kv4.3 preincubated with a dose of (3, 5, 8 and 10 nM) VER155008 for 24 h. (**E**) The hERG protein expression levels in cells co-transfected with Kv4.3 and hERG preincubated with 3 nM VER155008 were reduced to the same level as in cells transfected with hERG alone. (**F**) Absence or presence of hERG transfection did not affect the expression of Hsp70 induced by Kv4.3.

In our experiment, an increase in Hsp70 was observed in cells transfected with both hERG and Kv4.3 (Fig. 4A). Since previous study showed that overexpression of Hsp70 increased the expression of hERG protein^27^, we therefore hypothesized that Kv4.3 increased the hERG protein expression via upregulation of Hsp70. To test the hypothesis, we examined the expression of hERG in cells transfected with both hERG and Kv4.3, in the presence or absence of Hsp70 siRNA or VER155008, a novel adenosine-derived inhibitor of Hsp70^28,29^. The results showed that downregulation of Hsp70 using siRNA (Fig. 4B & C) or VER155008 (Fig. 4D and E) effectively abolished the elevation of matured hERG protein expression in the cells co-transfected with hERG and Kv4.3. In addition, as shown in Fig. 4F, absence or presence of hERG transfection did not affect the expression of Hsp70 induced by Kv4.3 transfection, further indicating that hERG itself has no role in the Hsp70 expression. In summary, these results suggest that Kv4.3 enhances hERG protein expression through Hsp70.

### Interactions between hERG and Kv4.3

**Figure 5:**
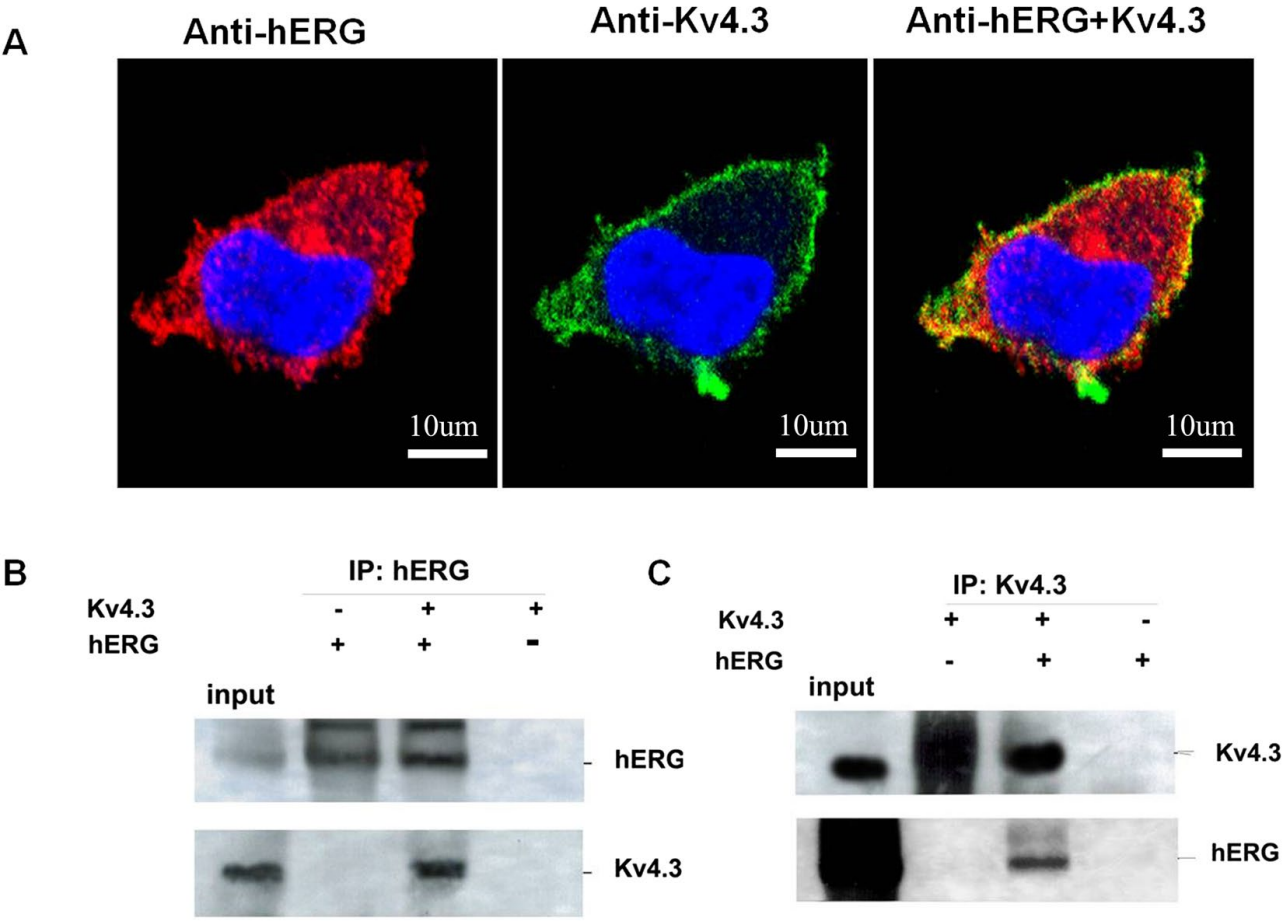
Interaction and complex formation between hERG and Kv4.3. (**A**) Red represents the immunofluorescence of secondary antibody to anti-hERG. Green represents the Kv4.3 signal. Yellow staining indicates co-localization of hERG and Kv4.3. Proteins were immunoprecipitated with (**B**) anti-hERG or (**C**) anti-Kv4.3 and immunoblotted with anti-hERG and anti-Kv4.3. Whole-cell lysates from cells co-transfected with Kv4.3 and hERG served as input control. The co-immunoprecipitation shows that hERG and Kv4.3 indeed interacted with each other.

Double immunofluorescent staining using anti-hERG and anti-Kv4.3 antibody revealed that hERG and Kv4.3 co-localize predominantly at the membrane (Fig. 5A). To assess the possible physical interaction between the two α subunits, immunoprecipitation experiments were carried out in 293 T cells transiently transfected with either hERG or Kv4.3, or co-transfected with hERG and Kv4.3. The whole-cell lysates from cells co-transfected with hERG and Kv4.3 served as an input control. Figure 5B shows that Kv4.3 was precipitated by anti-hERG antibody from the whole-cell lysate of cells co-transfected with hERG and Kv4.3. hERG proteins, both the 135 kDa and 155 kDa forms, were also detected in a reciprocal IP with the Kv4.3 antibody (Fig. 5C). These data showed that hERG co-localize and bound with Kv4.3.

### Interactions of Hsp70 with hERG and Kv4.3

**Figure 6:**
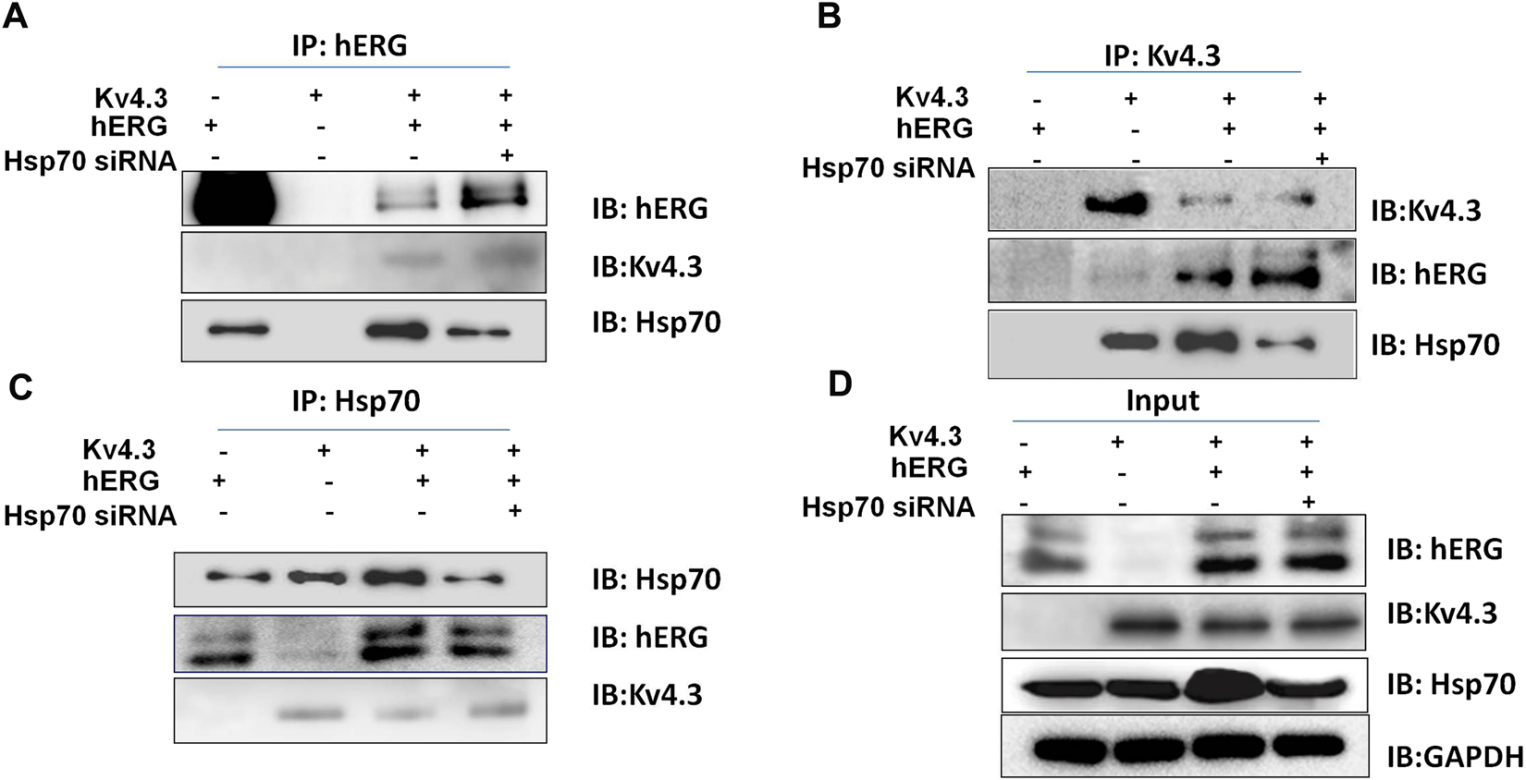
Hsp70 interacts and forms complexes with hERG and Kv4.3. Inhibiting Hsp70 protein expression did not affect the interaction between hERG and Kv4.3. Proteins were immunoprecipitated with (**A**) anti-hERG, (**B**) anti-Kv4.3, or (**C**) anti-Hsp70. (**D**) Whole-cell lysates from cells transfected with hERG or Kv4.3 alone, co-transfected with hERG /Kv4.3 or co-transfected with hERG/Kv4.3 and Hsp70 siRNA served as input control.

Because Hsp70 was reported to interact with hERG^27^ and the present study showed that hERG interacts with Kv4.3, we hypothesize that Hsp70 may form complexes with hERG and Kv4.3. We therefore perform immunoprecipitation using an anti-hERG antibody and found that, Hsp70 was precipitated from the whole-cell lysate of cells transfected with hERG and cells co-transfected with hERG and Kv4.3 (Fig. 6A). Further, Hsp70 was precipitated, with an anti-Kv4.3 antibody, from the whole-cell lysate of cells transfected with Kv4.3 and co-transfected with hERG and Kv4.3 (Fig. 6B). Of note, hERG and Kv4.3 were precipitated, with an anti-Hsp70 antibody, from the whole-cell lysate of all groups (Fig. 6C). Interestingly, down-regulation of Hsp70 using siRNA did not affect any of the immunoprecipitation results mentioned above. These data indicate that Hsp70 forms a complex with hERG and Kv4.3; however, absence of Hsp70 does not prevent the complex formation between hERG and Kv4.3.

### Interactions of Hsp90 with hERG and Kv4.3

**Figure 7:**
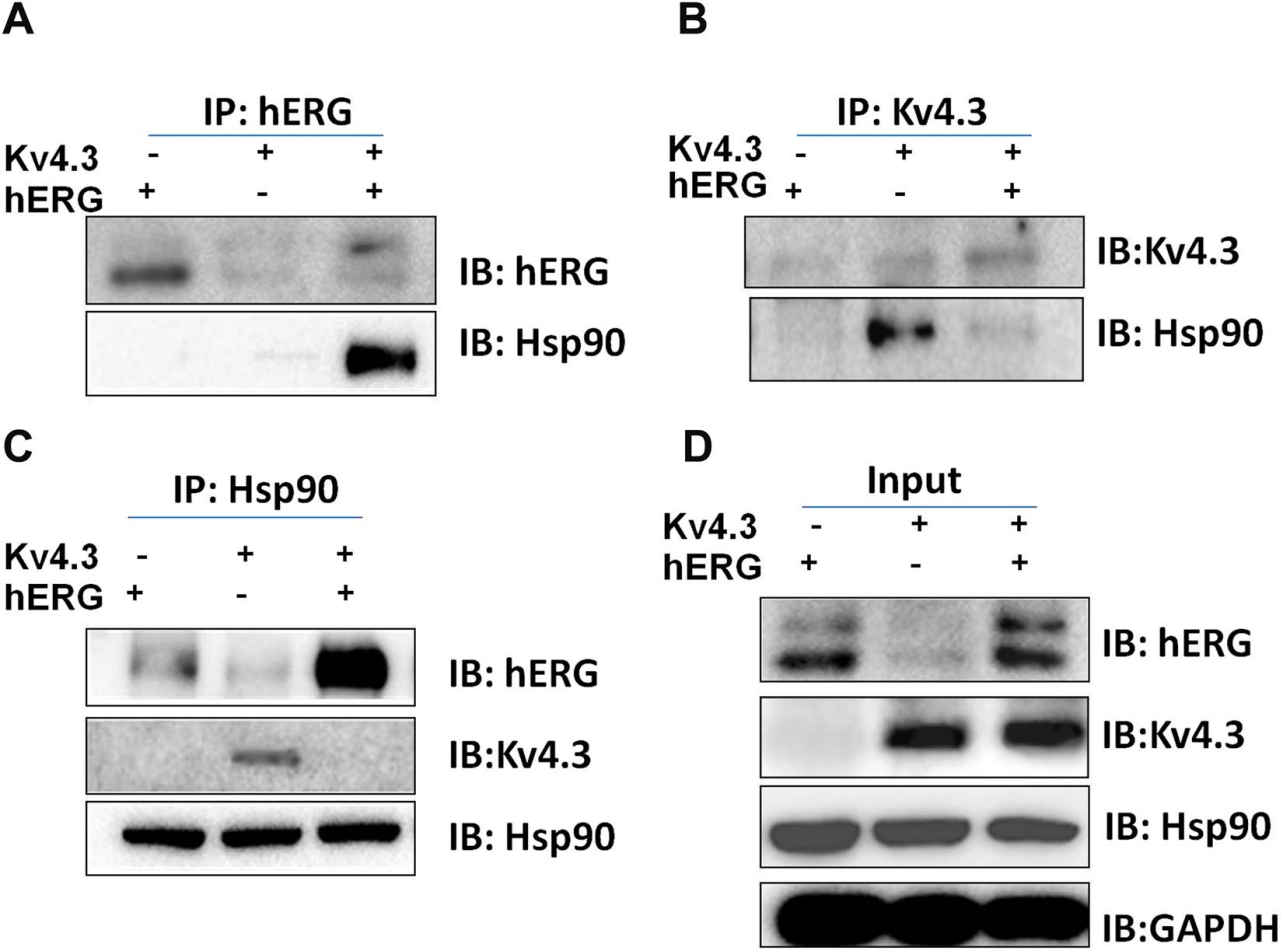
Hsp90 enhances hERG protein expression in cells co-transfected with hERG and Kv4.3. Proteins were immunoprecipitated with (**A**) anti-hERG, (**B**) anti-Kv4.3, or (**C**) anti-Hsp90. (**D**) Whole-cell lysates from cells transfected with hERG or Kv4.3 alone, or co-transfected with hERG/Kv4.3 served as input control. Co-immunoprecipitation showed that Hsp90 did not interact with hERG alone; however, Hsp90 interacted with the 135KD immature hERG in cells co-transfected with hERG and Kv4.3. Hsp90 interacted with Kv4.3 in cells transfected with Kv4.3 alone, but not in cells co-transfected with hERG and Kv4.3.

Hsp90 was also reported to enhance hERG protein expression^30^. However, our results showed that the protein expression of Hsp90 were not affected by hERG or hERG/Kv4.3 transfection (Fig. 7D). Interestingly, Hsp90 protein expression was precipitated, with an anti-hERG antibody, from the whole-cell lysate of hERG and Kv4.3 co-transfected cells, but not from the cells transfected with hERG alone (Fig. 7A). Conversely, Hsp90 protein expression was precipitated, with an anti-Kv4.3 antibody, from whole-cell lysate of cells transfected with Kv4.3 alone, but not from cells co-transfected with hERG and Kv4.3 (Fig. 7B). Immunoprecipitation with anti-Hsp90 showed that Hsp90 interacted with hERG in cells co-transfected with hERG and Kv4.3. However, Hsp90 only interacted with 135 kDa hERG and not with 155 kDa hERG (Fig. 7C).

## Discussion

In the present study, we have demonstrated physical interactions between Kv4.3 and hERG that were associated with increased hERG-protein expression and enhanced current density without altering the biophysical properties of hERG currents. On the other hand, co-expression with hERG did not alter the cellular distribution of Kv4.3 or the density or property of Kv4.3 currents.

hERG encodes the α subunit of I_Kr_. Kv4.3 is the α subunit of human I_to_. Our study indicates a novel potential molecular interaction between these two α subunits of hERG and Kv4.3. Kv4.3-induced hERG protein expression were paralleled by an increase in I_hERG_ density. In the presence of Kv4.3, I_hERG_ deactivation time constants were smaller, but no difference was found in the steady-state activation and inactivation. Also, the co-transfection of hERG showed no effect on the protein expression and the biophysical properties of Kv4.3.

Under physiological conditions, the wild type hERG exhibits two bands on Western blot analysis; this represents different stages of asparagine (Asn)-linked glycosylation^31^. The core-glycosylated, immature form is represented by a 135 kDa band and is present in the endoplasmic reticulum (ER); the fully glycosylated mature protein is observable as a 155 kDa band and represents hERG localizes either in the Golgi apparatus or on the cell surface^32,33^. The comparison of the intensity of these two bands provides a useful details regarding hERG trafficking. We found that the mature form of hERG (155 kDa) increased after the cells were co-transfected with Kv4.3. However, since transient transfection was used in our experiments, majority of the protein may still be within intracellular milieu (immature). These results were further verified by confocal imaging. In the presence of Kv4.3, more hERG protein localized on the surface of the cells, thus suggesting that Kv4.3 enhances hERG trafficking.

Ficker *et al*. showed that the cytosolic chaperones Hsp70 interacted with hERG during maturation^27^. The Hsp70 family, including stress-induced Hsp70 and constitutively expressed heat shock cognate protein (Hsc)70, was shown to interact with the core-glycosylated form of hERG^27,34^ and increases the levels of mature form of hERG^27^. Based on aforementioned studies, we hypothesize that Hsp70 may be involved in the maturation of hERG induced by Kv4.3. Indeed, we found that Hsp70 protein expressions were increased in cells co-transfected with hERG and K4.3. The upregulation of hERG expression by Kv4.3 is dependent on Hsp70, as Hsp70 siRNA could prevent the Kv4.3-induced hERG expression. Similar result was obtained when Hsp70 inhibitor, VER155008, was used. These results suggest that Kv4.3 enhances hERG protein trafficking through Hsp70.

Since the confocal image demonstrated that hERG and Kv4.3 co-localized on cell surface, we further investigated the interaction between hERG and Kv4.3 using immunoprecipitation. The result showed that hERG and Kv4.3 bind to each other and form a complex. Both mature and non-mature forms of hERG co-immunoprecipitated with Kv4.3.

Interactions between hERG and potential α and β subunits have been suggested. For instances, KvLQT1 was shown to interact with hERG to modulate hERG localization and current densities^20^. KvLQT1 could also interact with hERG at the C-terminal cytoplasmic tails of hERG that contained cyclic nucleotide binding domain^20^. MiRP1 is also believed to interact with hERG at the C-terminal region^35,36^. It is known that long-QT syndrome type 2 (LQT2) mutations in the C-terminal tail affect trafficking of hERG from the ER to the cell surface^37–39^. A number of the C-terminal interacting proteins such as GM130 and 14–3–3 have been identified in playing potential roles in the trafficking of hERG to the cell surface^40–42^. However, in our study, we did not determine which domain of hERG interacted with Kv4.3.

We observed that Kv4.3 interacted with hERG and increased hERG expression through Hsp70. It has been reported that hERG formed complexes with Hsp70^27^. Therefore, we tested if Hsp70 formed complexes with hERG and Kv4.3, and if Hsp70 mediated the interactions between hERG and Kv4.3. Our data demonstrated that both Kv4.3 and hERG interacted with Hsp70, suggesting that Hsp70 may form complexes with hERG and Kv4.3. However, although downregulation of Hsp70 decreased hERG protein expression in cells co-transfected with Kv4.3 and hERG, Hsp70 did not play a critical role in the complex formation between hERG and Kv4.3. These data indicate that Hsp70 increases hERG protein expression and forms complexes with hERG via two independent mechanisms.

Hsp90 only interacted with Kv4.3, and not with hERG when expressed alone (Fig. 7). However, Hsp90 preferably interacted with hERG in cells co-transfected with hERG and Kv4.3 and enhanced the expression of 135 kDa hERG. These results suggest that Hsp90 plays a very complicated role in the intereaction between hERG and Kv4.3, which warrants further study.

There are a few limitations in the present study. In all experiments, hERG and Kv4.3 were transiently transfected in cells; therefore, the immature form of hERG had higher band intensity than the mature form. Our studies were performed primarily in HEK293T cells. It remained to be determined whether the interactions that we observed were specific to this cell line. The association between hERG and Kv4.3 was not verified in cardiac myocytes or animal model. Therefore caution should be taken to extrapolate the findings to cardiac myocytes because cardiac myocytes express many other ion channels which may increase or decrease the interaction between hERG and Kv4.3.

## Conclusion

In conclusion, our finding reveals the interaction between the α subunits of I_Kr_ hERG and I_to_ Kv4.3. Kv4.3 modulates hERG current density by promoting hERG protein expression and localization via Hsp70. Kv4.3 and hERG co-localize with each other, and form complexes with Hsp70 in HEK293T cells. The physical and functional interactions between the hERG and Kv4.3 enrich the theory of “repolarization reserve”.

## Materials and Methods

### Cell culture and transfection

hERG cDNA and Kv4.3 cDNA were provided by Dr. Silvia G. Priori (University of Pavia, Pavia, Italy). Human embryonic kidney 293 (HEK293T) were cultured at 37 °C with 5% CO_2_ in DMEM medium (Invitrogen) supplemented with 10% heat-inactivated fetal bovine serum (Gibco), 100 units/ml penicillin, and 100 µg/ml streptomycin (Invitrogen). HEK293T cells were transfected transiently or stably with these constructs using a lipofectamine method as described previously^20^. After the transient transfection, HEK293T cells were studied at 48 hours. When the transiently transfected cells were used for patch clamp experiments, CD8 cDNA was co-transfected to be used as a reporter gene (Invitrogen). CD8 (1 µg) was co-transfected with hERG cDNA (1 µg) or Kv4.3 cDNA (1 µg). CD8-positive cells were identified using Dynabeads (Dynal, M-450 CD8). Cells were harvested between 48–72 hours after the transfection.

### Patch Clamp Experiments

The HEK293T cells were bathed in the solution containing (in mmol/L) NaCl 140, KCl 4, CaCl_2_ 2, MgCl_2_ 1, HEPES 10 and glucose 5, adjusted to pH 7.4 with NaOH. The current was recorded by the whole cell patch-clamp technique using a MultiClamp 700B amplifier (Axon Instruments, USA). All signals were acquired at 5 kHz (Digidata 1322 A, Axon Instruments, USA). Patch pipettes were pulled from borosilicate glass on a P-97 horizontal puller (Sutter Instruments, USA). The electrodes had a resistance of 2–3 MΩ. In order to record the K^+^ currents, pipettes were filled with (in mmol/L): K-aspartame acid 140, MgATP 4, MgCl_2_ 1, EGTA 10, GTP 0.1, HEPES 10 and adjusted to pH 7.3 with KOH.

I_to_ was elicited by a 300 ms depolarizing pulse from a holding potential of −80 mV to a testing potential of +70 mV and a conditioning test of −40 mV for 25 ms to eliminate the sodium current. This current was blocked by 5 nM 4-aminopyridine (4-AP). I_hERG_ was investigated by applying voltage commands of 5000 ms duration from a holding potential of −80 mV to a range of test potentials from −70 mV to +50 mV, followed by a repolarizing step to −40 mV for 3000 ms to elicit tail currents. This current was significantly inhibited after exposure to 5 nM dofetilide. Average I/V relationships were used to compare current densities following the normalization of current traces to individual cell capacitance measurements. Steady-state I/V relationships were measured at the end of the test pulse.

### Immunofluorescence and Confocal Microscopy

For immunofluorescent studies, transiently transfected HEK293T cells were grown on glass bottom cell culture dishes for 24 hours. Cells were then fixed (10 min) with 5% paraformaldehyde (Sigma), washed 3 times (5 min) with a phosphate-buffered saline, blocked with a 3% bovine serum albumin (Sigma), and then permeabilized with 0.1% Triton X-100 (Sigma) for 10 minutes. Cells were incubated overnight (4 °C) with primary antibodies (anti-hERG Alomone, rabbits,) at 1:800 dilution followed by three 5-minutes washes with phosphate-buffered saline. Next, cells were incubated with Alexa Fluor 555 donkey anti-rabbit secondary antibody (Invitrogen) for 45 minutes at room temperature. After being washed, the cells were treated with 4′6-diamidino-2-phenylindole (DAPI; Sigma–Aldrich) for 5 minutes and analyzed under a confocal microscope.

### Western Blot Analysis and Co-immunoprecipitation (co-IP)

Whole cell proteins from HEK293T cells that expressed various channels were used for analysis. Proteins were separated on 8% or 12% SDS-polyacrylamide electrophoresis gels, transferred onto nitrocellulose membrane, and blocked for 1 hour with 5% nonfat milk. The blots were incubated with the primary antibody for 24 hours at 4 °C and then incubated with a horseradish peroxidase-conjugated secondary antibody. GAPDH expression was used as loading controls. The blots were visualized with Fujifilm using the ECL detection kit (GE Healthcare). For immunoprecipitation, a whole cell protein (0.5 mg) or cell surface protein was incubated with the appropriate primary antibody overnight at 4 °C and then precipitated with protein A/G plus agarose beads (Santa Cruz) for 4 hours at 4 °C. The beads were washed three times with an ice-cold radio immune precipitation assay lysis buffer, resuspended in 2 mL sample buffer, and boiled for 5 minutes. The samples were centrifuged at 20,000 g for 5 minutes, and the supernatants were collected and analyzed using Western blot.

### Data Availability

The datasets generated during and/or analyzed during the current study are available from the corresponding author on reasonable request.

## References

[1] Greenstein JL, Wu R, Po S, Tomaselli GF, Winslow RL (2000). Role of the calcium-independent transient outward current I(to1) in shaping action potential morphology and duration. Circ Res.

[2] Niwa N, Nerbonne JM (2010). Molecular determinants of cardiac transient outward potassium current (I(to)) expression and regulation. J Mol Cell Cardiol.

[3] Sanguinetti MC, Jurkiewicz NK (1990). Two components of cardiac delayed rectifier K+ current. Differential sensitivity to block by class III antiarrhythmic agent. s. J Gen Physiol.

[4] Balser JR, Bennett PB, Roden DM (1990). Time-dependent outward current in guinea pig ventricular myocytes. Gating kinetics of the delayed rectifier. J Gen Physiol.

[5] Roden DM (1998). Taking the “idio” out of “idiosyncratic”: predicting torsades de pointes. Pacing Clin Electrophysiol.

[6] Varro A (2000). The role of the delayed rectifier component IKs in dog ventricular muscle and Purkinje fibre repolarization. J Physiol.

[7] Biliczki P, Virag L, Iost N, Papp JG, Varro A (2002). Interaction of different potassium channels in cardiac repolarization in dog ventricular preparations: role of repolarization reserve. Br J Pharmacol.

[8] Rudy Y (2007). Modelling the molecular basis of cardiac repolarization. Europace.

[9] Zeng J, Laurita KR, Rosenbaum DS, Rudy Y (1995). Two components of the delayed rectifier K + current in ventricular myocytes of the guinea pig type. Theoretical formulation and their role in repolarization. Circ Res.

[10] Roden DM (2008). Repolarization reserve: a moving target. Circulation.

[11] Kass RS, Freeman LC (1993). Potassium channels in the heart Cellular, molecular, and clinical implications. Trends Cardiovasc Med.

[12] MacKinnon R (1991). Determination of the subunit stoichiometry of a voltage-activated potassium channel. Nature.

[13] Sanguinetti MC, Jiang C, Curran ME, Keating MT (1995). A mechanistic link between an inherited and an acquired cardiac arrhythmia: HERG encodes the IKr potassium channel. Cell.

[14] Abbott GW, Ramesh B, Srai SK (2008). Secondary structure of the MiRP1 (KCNE2) potassium channel ancillary subunit. Protein Pept Lett.

[15] Abbott GW, Goldstein SA, Sesti F (2001). Do all voltage-gated potassium channels use MiRPs?. Circ Res.

[16] Nerbonne JM, Kass RS (2005). Molecular physiology of cardiac repolarization. Physiol Rev.

[17] Zhang M, Jiang M, Tseng G (2001). N. minK-related peptide 1 associates with Kv4.2 and modulates its gating function: potential role as beta subunit of cardiac transient outward channel?. Circ Res.

[18] Roepke TK (2008). Targeted deletion of kcne2 impairs ventricular repolarization via disruption of I(K, slow1) and I(to, f). FASEB J.

[19] Delpon E (2008). Functional effects of KCNE3 mutation and its role in the development of Brugada syndrome. Circ Arrhythm Electrophysiol.

[20] Ehrlich JR (2004). KvLQT1 modulates the distribution and biophysical properties of HERG. A novel alpha-subunit interaction between delayed rectifier currents. J Biol Chem.

[21] Wu J (2010). KCNE2 modulation of Kv4.3 current and its potential role in fatal rhythm disorders. Heart Rhythm.

[22] Coronel R (2013). Electrophysiological changes in heart failure and their implications for arrhythmogenesis. Biochim Biophys Acta.

[23] Janse MJ (2004). Electrophysiological changes in heart failure and their relationship to arrhythmogenesis. Cardiovasc Res.

[24] Kaab S (1998). Molecular basis of transient outward potassium current downregulation in human heart failure: a decrease in Kv4.3 mRNA correlates with a reduction in current density. Circulation.

[25] Gomez JF, Cardona K, Romero L, Ferrero JM, Trenor B (2014). Electrophysiological and structural remodeling in heart failure modulate arrhythmogenesis. 1D simulation study. PLoS One.

[26] Walmsley J (2013). mRNA expression levels in failing human hearts predict cellular electrophysiological remodeling: a population-based simulation study. PLoS One.

[27] Ficker E, Dennis AT, Wang L, Brown AM (2003). Role of the cytosolic chaperones Hsp70 and Hsp90 in maturation of the cardiac potassium channel HERG. Circ Res.

[28] Massey AJ (2010). A novel, small molecule inhibitor of Hsc70/Hsp70 potentiates Hsp90 inhibitor induced apoptosis in HCT116 colon carcinoma cells. Cancer Chemother Pharmacol.

[29] Wen W, Liu W, Shao Y, Chen L (2014). VER-155008, a small molecule inhibitor of HSP70 with potent anti-cancer activity on lung cancer cell lines. Exp Biol Med (Maywood).

[30] Iwai C (2013). Hsp90 prevents interaction between CHIP and HERG proteins to facilitate maturation of wild-type and mutant HERG proteins. Cardiovasc Res.

[31] Zhou Z (1998). Properties of HERG channels stably expressed in HEK 293 cells studied at physiological temperature. Biophys J.

[32] Petrecca K, Atanasiu R, Akhavan A, Shrier A (1999). N-linked glycosylation sites determine HERG channel surface membrane expression. J Physiol.

[33] Gong Q, Anderson CL, January CT, Zhou Z (2002). Role of glycosylation in cell surface expression and stability of HERG potassium channels. Am J Physiol Heart Circ Physiol.

[34] Walker VE, Atanasiu R, Lam H, Shrier A (2007). Co-chaperone FKBP38 promotes HERG trafficking. J Biol Chem.

[35] Cui J (2001). Analysis of the cyclic nucleotide binding domain of the HERG potassium channel and interactions with KCNE2. J Biol Chem.

[36] Schlichter LC (2014). Regulation of hERG and hEAG channels by Src and by SHP-1 tyrosine phosphatase via an ITIM region in the cyclic nucleotide binding domain. PLoS One.

[37] Zhou Z, Gong Q, Epstein ML, January CT (1998). HERG channel dysfunction in human long QT syndrome. Intracellular transport and functional defects. J Biol Chem.

[38] Kupershmidt S (2002). Defective human Ether-a-go-go-related gene trafficking linked to an endoplasmic reticulum retention signal in the C terminus. J Biol Chem.

[39] Anderson CL (2014). Large-scale mutational analysis of Kv11.1 reveals molecular insights into type 2 long QT syndrome. Nat Commun.

[40] Roti EC (2002). Interaction with GM130 during HERG ion channel trafficking. Disruption by type 2 congenital long QT syndrome mutations. Human Ether-a-go-go-Related Gene. J Biol Chem.

[41] Zhang KP, Yang BF, Li BX (2014). Translational toxicology and rescue strategies of the hERG channel dysfunction: biochemical and molecular mechanistic aspects. Acta Pharmacol Sin.

[42] Kagan A, Melman YF, Krumerman A, McDonald TV (2002). 14-3-3 amplifies and prolongs adrenergic stimulation of HERG K+ channel activity. EMBO J.

